# A Case of Urachal Carcinoma of the Abdominal Wall in a Kidney Transplant Recipient

**DOI:** 10.1155/2017/3919635

**Published:** 2017-05-04

**Authors:** Takuya Yamazaki, Yasushi Nagaba, Yoshitaka Shimada, Yoshinori Taoka, Satoru Minamida, Dai Koguchi, Masahiro Hagiwara, Sho Watanuki, Hide Nagaba, Kazunari Yoshida, Yasuo Takeuchi

**Affiliations:** ^1^Department of Nephrology, Kitasato University Medical Center, Saitama, Japan; ^2^Department of Urology, Kitasato University Medical Center, Saitama, Japan; ^3^Department of Urology, Kitasato University School of Medicine, Kanagawa, Japan; ^4^Department of Nephrology, Kitasato University School of Medicine, Kanagawa, Japan

## Abstract

Urachal carcinoma is an extremely rare malignant tumor arising from the urachus in the fetus. We report a patient who developed urachal carcinoma 18 years after kidney transplantation. A 59-year-old man was admitted because of abdominal pain and massive ascites. He had undergone kidney transplantation 18 years earlier and had end-stage renal disease requiring dialysis. Abdominal CT showed massive ascites and an abdominal wall cystic mass separated from the peritoneal cavity. Hemodialysis was started, and paralytic ileus was diagnosed and treated. His ileus symptoms improved temporarily, but he died of myocardial infarction. An autopsy was performed, which revealed cystadenocarcinoma in the abdominal wall mass, leading to a diagnosis of urachal carcinoma.

## 1. Introduction

Advances in immunosuppressive therapy have improved the outcome of kidney transplantation and dramatically increased the number of patients with long-term graft survival. However, the incidence of complications involving organs other than the transplanted kidney increases according to the number of years after transplantation. Transplant-associated malignant tumors are the leading cause of death in patients who have survived at least 10 years after transplantation. Although the development of malignant tumors is influenced by the type of immunosuppressive drugs used, skin cancers and malignant lymphomas are, in general, common. The reported incidence of malignant tumors in urology ranges from 16.0 to 28.4% [1, 2]. We report a case of urachal carcinoma which developed 18 years after kidney transplantation.

## 2. Case Report

**Figure 1:**
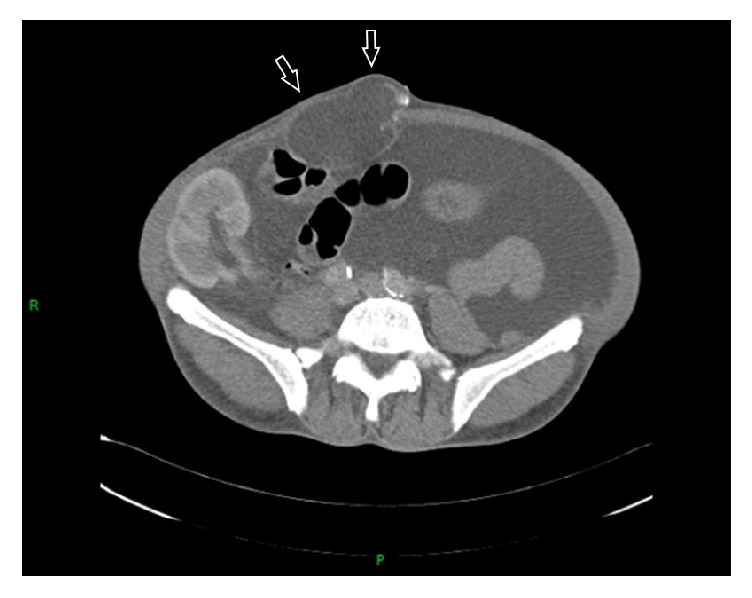
Abdominal CT on admission showed massive ascites and a cystic mass in the abdominal wall (arrows).

**Figure 2:**
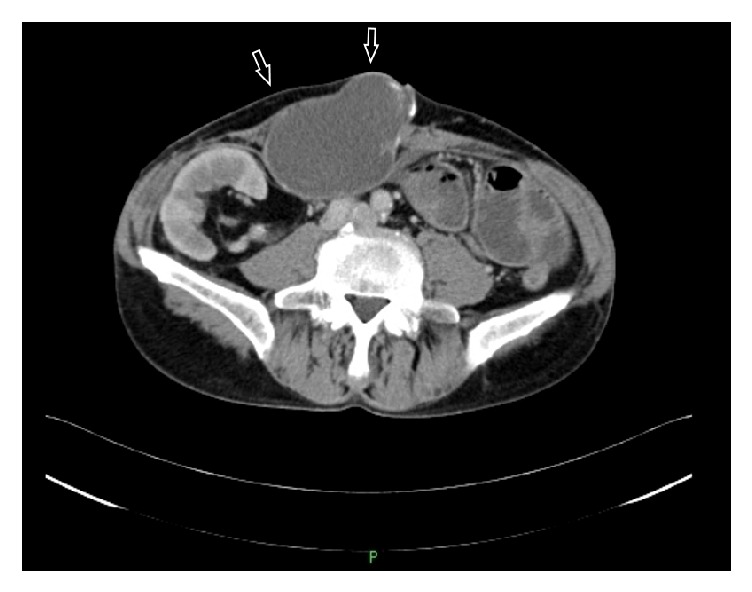
Abdominal CT at 4 weeks after dialysis initiation showed a marked decrease in the volume of ascitic fluid, with no change in the abdominal wall mass (arrows).

A 59-year-old man was admitted because of anorexia and massive ascites. He had received peritoneal dialysis for chronic renal failure for 4 years, undergone cadaveric kidney transplantation 18 years earlier, and had been withdrawn from peritoneal dialysis. He had received immunosuppressive agents, such as tacrolimus, azathioprine, and prednisolone. The transplanted kidney was functioning well. For about 10 years after kidney transplantation, the general condition, including renal function, was stable, but the renal function gradually worsened over a period of 3 years before admission. On admission, the serum creatinine level was high, at 8.2 mg/dL. There were massive ascites, and a mass of 10 cm in diameter was observed in the abdominal wall. The abdomen was distended. He vomited repeatedly and was unable to eat. He was diagnosed with paralytic ileus, intubated with a nasogastric tube, and allowed to eat nothing by mouth. A central venous catheter was inserted, and a high-calorie infusion was administered for systemic management of the condition. The ascitic fluid was found to be transudative, its culture was negative for bacteria, and its cytology was negative for malignant cells. Blood tumor markers were measured: CEA was slightly elevated to 12.6 ng/mL (normal, ≤5.0 ng/mL), and CA19-9 was 34.2 U/mL (normal, ≤37.0 U/mL). Abdominal CT showed massive ascites and a cystic mass in the abdominal wall behind the navel (Figure 1). The cystic mass had a slightly inhomogeneous density and did not communicate with the peritoneal cavity. Contrast-enhanced abdominal CT, performed to determine the underlying cause of the ileus, revealed multiple small-bowel strictures probably due to adhesions and compression. Due to the development of uremic symptoms, an internal shunt was constructed, and hemodialysis was started on the 21st hospital day. Hemodialysis induced a decrease in the ascitic fluid volume but did not diminish the size of the abdominal wall mass (Figure 2). On the 52nd hospital day, hemodialysis was stable, and the patient recovered to the extent that he could eat; therefore, he was discharged. He was scheduled for elective resection of the abdominal wall cystic mass.

After hospital discharge, he received maintenance dialysis three times weekly, and his course on hemodialysis was uneventful. Three months after discharge, he visited the emergency room because of chest pain. He was in cardiopulmonary arrest on arrival, and resuscitation maneuvers were performed. His heartbeat was temporarily restored; however, 3 days later, he developed cardiopulmonary arrest again and was confirmed dead. An autopsy was performed.

## 3. Autopsy Findings

**Figure 3:**
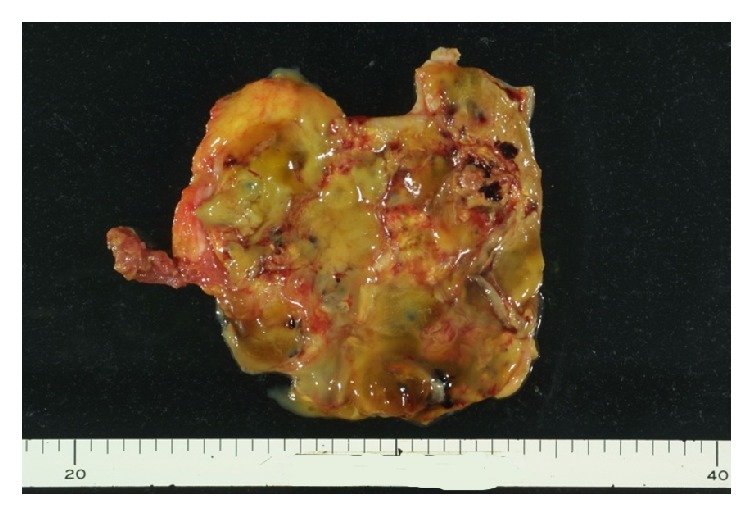
Abdominal wall mass removed at autopsy.

**Figure 4:**
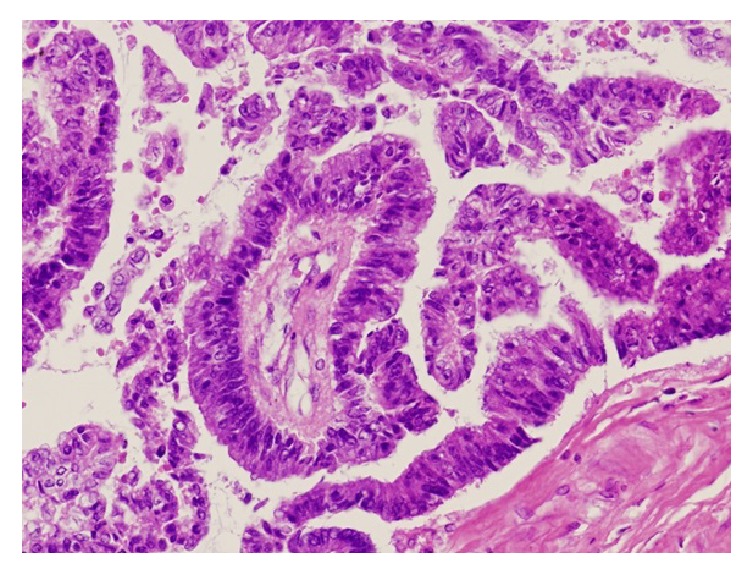
Histological examination showed low-grade malignant mucinous cystadenocarcinoma.

After death, an autopsy was performed. The heart showed multiple subendocardial infarcts, suggesting that the immediate cause of death was acute myocardial infarction. A mass was noted in the subcutaneous tissue of the abdominal wall around the navel (Figure 3). The mass measured 13 × 9 × 6 cm, weighed 435 g, and was histologically diagnosed as low-grade malignant mucinous cystadenocarcinoma (Figure 4). This tumor was not located in the bladder, but in the abdominal wall at a distance from the bladder. Adenocarcinoma in the abdominal wall needs to be differentiated from colorectal carcinoma. In this patient, the tumor and colon were clearly separate from each other, and no cancer tissue was observed in the colon. A careful search of other organs failed to detect the primary lesion for adenocarcinoma. The location and histological type of the mass suggested that it arose from the urachal remnant, and it was diagnosed as urachal carcinoma. This tumor was located in the abdominal wall at a distance from the bladder, where no malignant tissue was seen. There were ileoperitoneal adhesions in the left upper abdomen, and the surrounding peritoneum showed marked fibrous thickening, indicating peritoneal sclerosis, with no evidence of peritoneal malignancy.

## 4. Discussion

The risk of developing malignant tumors after renal transplantation increases with time after kidney transplantation and with the patient's age. In particular, in patients aged 65 or older, the relative risk of developing malignant tumors reportedly increases to five times that in healthy individuals. Immunosuppressive agents, viral infections, race, and genetic factors are considered to be involved in the increase in cancer risk after renal transplantation, and the types of malignant tumors reportedly differ depending on the immunosuppressive agent used. Among the urological malignant tumors, renal cell carcinoma is the most common, and there are many reports of bladder cancer [1, 2]. Urachal carcinoma is a rare cancer arising from the remnant epithelial tissue of the urachus and reportedly has a predilection for the dome of the bladder, but, in some patients, it arises from the urachal remnant of the abdominal wall. To date, only one patient has been reported who developed urachal carcinoma in the bladder after kidney transplantation [3].

During fetal life, the urachus communicates with the cloaca and extends from the bladder to the navel; however, in adults, it has atrophied to become a cord-like structure. Part of the urachal epithelium may remain. Urachal carcinoma was first reported in 1863. The incidence of urachal carcinoma as a bladder cancer is 0.35–0.7%. To date, more than 1,000 cases have been reported in the world literature. Urachal carcinoma frequently (90%) arises in the vicinity of the bladder, but there are a few reports of it arising in the abdominal wall. More than 80% of urachal carcinomas are adenocarcinoma. They recur locally at a high rate and are associated with a poor prognosis, with a 5-year survival rate of up to 50%. Urachal carcinoma arising in the bladder causes symptoms similar to those caused by usual bladder cancer, but that arising in the abdominal wall is often difficult to diagnose and does not cause uniform symptoms [4, 5].

The present patient had a 4-year history of peritoneal dialysis and developed urachal carcinoma with massive ascites 18 years after kidney transplantation. His history of receiving peritoneal dialysis therapy may have contributed to the development of massive ascites. Pathologically, part of the peritoneum showed sclerotic changes, suggesting the presence of a cause-effect relationship between peritoneal dialysis and urachal carcinoma of the abdominal wall. The association between the development of urachal carcinoma and peritoneal dialysis and kidney transplantation is unclear, because urachal carcinoma itself is an extremely rare malignant tumor, because there has been only one reported case of posttransplant urachal carcinoma and because no case of urachal carcinoma developing after peritoneal dialysis has been reported to date.

## 5. Conclusions

We report the case of a 59-year-old man who underwent renal transplantation after being maintained on peritoneal dialysis for 4 years and was found to have developed urachal carcinoma in the peritoneal wall after the resumption of peritoneal dialysis. He initially presented with massive ascites and followed a peculiar course. This was probably due partly to the fact that he underwent kidney transplantation as well as peritoneal dialysis. The urachal carcinoma in this case was too difficult to definitively diagnose before death.
